# Safety of Credelio Quattro™ (lotilaner, moxidectin, praziquantel, and pyrantel chewable tablets) in dogs infected with adult heartworms (*Dirofilaria immitis*)

**DOI:** 10.1186/s13071-025-06732-z

**Published:** 2025-04-14

**Authors:** Kari L. Riggs, Deanna Haney, Scott Wiseman

**Affiliations:** 1 Elanco Animal Health, 2500 Innovation Way, Greenfield, IN 46140 USA; 2 Elanco Animal Health, Form 2, Bartley Way, Bartley Wood Business Park, Hook, RG27 9XA UK

**Keywords:** Credelio Quattro, Lotilaner, Moxidectin, Praziquantel, Pyrantel, Safety, Heartworm-positive, Canine

## Abstract

**Background:**

Credelio Quattro (lotilaner, moxidectin, praziquantel, and pyrantel chewable tablets) is a novel endectocide for monthly oral administration in dogs. The safety of Credelio Quattro was investigated in dogs with pre-existing patent heartworm (*Dirofilaria immitis*) infections. Heartworm preventive products are tested in heartworm-positive dogs as rapid microfilarial and adult worm death can lead to serious clinical reactions, including death.

**Methods:**

This was a gender-stratified, randomized, placebo-controlled, blinded, parallel group design study. Prior to study, dogs were surgically implanted with 10 male and 10 female adult *D. immitis* worms (Georgia III isolate). After confirming a patent infection, dogs were randomized into three groups (placebo control, 1×, or 3× the maximum recommended labeled dose of Credelio Quattro) consisting of eight dogs each. Treatment was administered on three consecutive monthly occasions. The assessment of safety was based on body weight, physical examinations, clinical observations on the days of dosing, general health observations, microfilariae (MF) counts, and *D. immitis* antigen testing. On the last day of study, the heart, lungs, and pleural and peritoneal cavities were examined for adult *D. immitis* worms.

**Results:**

Credelio Quattro was well tolerated. Emesis occurred in the 3× group only. Diarrhea was observed in all groups at various times throughout the study. Owing to the timing of events relative to dosing, emesis and diarrhea were possibly related to treatment; however, all dogs recovered quickly and without treatment. No signs of avermectin toxicity or hypersensitivity reactions were observed in any dog. Compared with control, Credelio Quattro reduced the concentration of circulating MF on study day 1 by 38.8% for the 1× group and significantly reduced MF by 73.3% for the 3× group. MF reduction remained significant for both groups at all subsequent time points.

**Conclusions:**

Credelio Quattro, when administered at 1× and 3× the maximum recommended label dose, was well tolerated following three consecutive monthly administrations to heartworm-positive dogs. Although Credelio Quattro caused a rapid reduction in microfilaria counts, no adverse effects related to microfilaria reduction were observed, and there was no effect on adult worms in this study.

**Graphical abstract:**

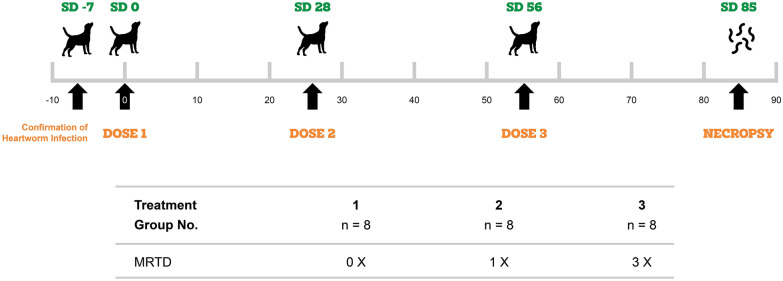

## Background

Heartworm (*Dirofilaria immitis*) disease in dogs is one of the most prevalent parasitic diseases worldwide and is well documented owing to its importance in companion animal health. It is a potentially fatal parasitic filarial disease causing severe lung pathology and morbidity in infected dogs, leading to acute disease, shortening of the animal’s life expectancy, and potentially death [1]. Although heartworm disease can be successfully prevented by systematic administration of chemoprophylactic drugs in dogs, the disease prevalence continues to increase and spread [2–4]. Annually, it is reported that over 100,000 dogs in the USA alone have heartworm disease [5]. In a retrospective study based on heartworm antigen testing reported to the Companion Animal Parasite Council (CAPC), it was found that two thirds of the dogs in the USA do not receive heartworm prophylaxis every year [3].

Prophylactic drugs, diethylcarbamazine (DEC) [6] and macrocyclic lactones (ML), target the heartworm lifecycle’s vulnerable third stage (L3) and early fourth stages (L4) and ameliorate the disease progression. DEC first entered the animal health market in the 1960s and was used as a heartworm preventive agent [7, 8]. Unfortunately, administering DEC to dogs as part of the preventive regimen without prior testing for microfilaremia induces fatal adverse reactions [9]. The exact mechanism of DEC-induced adverse reactivity in microfilaremic dogs is not known, but there is evidence that the host response to substances released from dead microfilariae (MF) causes hepatic vein constriction, leading to hepatic venous congestion and hypovolemic shock [10]. Currently, MLs are the only commercially available class of molecules approved for the prevention of heartworm disease in dogs [11]. The MLs are a family of compounds derived from soil-dwelling fungi, *Streptomyces*, which consist of avermectins and milbemycins. The commercially available avermectins are ivermectin, abamectin, doramectin, eprinomectin, and selamectin, whereas nemadectin, milbemycin oxime, and moxidectin are classified under milbemycins [12, 13]. ML formulations have been successfully utilized for the prevention of heartworm disease since the Food and Drug Administration’s (FDA) approval of ivermectin in 1987 (Heartgard-30^®^, Boehringer Ingelheim, Duluth, GA, USA) and milbemycin oxime (Interceptor^®^, Elanco, Greenfield, IN, USA) in 1990 [14].

In the past decade, studies have shown evidence of adulticidal properties of long-term administration of MLs in combination with doxycycline [15–17]; however, melarsomine dihydrochloride is the only approved adulticidal drug on the market [11]. The American Heartworm Society (AHS) currently recommends the use of an ML in combination with doxycycline prior to administration of melarsomine treatment to reduce the endosymbiont *Wolbachia* in adult stages and eliminate circulating microfilariae [11, 18]. In addition, ML administration prior to adulticidal melarsomine treatment eliminates migrating larval tissue stages of *D. immitis* to prevent the development of these larvae into the adult stages [11].

All MLs have exhibited microfilaricidal properties to varying degrees, but at present there are very few FDA-approved microfilaricide products in the USA, all containing 10 mg/kg imidacloprid and 2.5 mg/kg moxidectin administered topically, namely Advantage Multi^®^ (Elanco, Greenfield, IN) and two generic drug approvals (IMOXI^™^ (Vetoquinol, Fort Worth, TX) and PARASEDGE™ Multi (Virbac, Fort Worth, TX)) [6]. As per the AHS recommendation, dogs should be tested prior to heartworm prophylaxis for existing infection [11] to prevent potential adverse events following destruction of MF. In the USA, the FDA requires a thorough investigation and clear guidance for clinicians on the effects of any new heartworm prophylactic formulations, in microfilaremic dogs [14].

A key factor in the successful prevention of heartworm disease is owner compliance with prophylaxis during the exposure season currently recommended by the European Scientific Counsel Companion Animal Parasites (ESCCAP) [19] or year-round prophylaxis [20], as recommended by the AHS [11] and the Companion Animal Parasite Council [5]. Improper compliance with recommended prophylaxis regimens of MLs for heartworm could lead to a lack of efficacy (LOE) [3, 20] and, even worse, repeated administration of MLs to dogs with persistent heartworm infection may lead to genetic selection of ML-resistant strains of heartworm as in the Lower Mississippi Delta region [21], which is a growing concern in the USA.

MLs target the glutamate-gated chloride ion channels (GluCls) in invertebrates and filarial nematodes. Among the ML molecules, moxidectin has the unique characteristic of being highly lipophilic and has higher lipophilicity log*P* (5.4) as compared with ivermectin (4.3) [22]. This pharmacokinetic property of moxidectin leads to its higher tissue distribution compared with ivermectin and longer elimination half-life. In other words, it acts as a slow-release repository in the tissues and can target the tissue migratory L3 and L4 life stages [2]. Another unique feature of moxidectin is the way that it binds to GluCls, as it has subtle structural differences making the binding to nematode GluCls different to that of the other MLs, hence reducing the risk of drug resistance to moxidectin developing [2]. Moxidectin has shown evidence of superior prophylaxis against ML-resistant strains compared with other MLs, with increasing dose and frequency of administration providing the highest efficacy [23, 24].

Lotilaner is an ectoparasiticide from the new chemical class of isoxazolines [25]. Members of this class are potent inhibitors of insect ligand-gated chloride channels (LGCC) [26]. Lotilaner is available as a mono-use drug product (Credelio^®^, Elanco Animal Health) against ticks and fleas [27–29] and as a combination with milbemycin oxime (Credelio Plus^®^, Elanco Animal Health). Pyrantel is a member of the tetrahydropyrimidine family, with anthelmintic activity against a broad spectrum of both adult and immature endoparasites; it was first introduced into the healthcare market by Pfizer in the 1960s [30]. Praziquantel, synthesized by Merck and Bayer in 1972, was developed as a novel anthelminthic with a broad spectrum of action against parasitic trematodes and cestodes [31, 32]. Pharmacokinetic studies on praziquantel have shown rapid absorption when administered orally, and half-life varies between 0.1 h and 0.3 h [31]. Praziquantel is effective against both juvenile and adult cestodes in dogs [33].

A new chewable tablet containing moxidectin with anthelmintics, praziquantel and pyrantel, and lotilaner was developed to provide broad-spectrum efficacy against most endo- and ectoparasites in dogs, including prevention of *D. immitis* infection when administered once monthly. The study presented here aimed to evaluate the safety of Credelio Quattro (lotilaner, moxidectin, praziquantel, and pyrantel chewable tablets) when administered once a month orally at the 0×, 1×, and 3× maximum recommended therapeutic dose (MRTD) for 3 consecutive months to dogs with patent heartworm infection.

## Methods

The use of animals and all animal procedures were approved by the test facility’s Institutional Animal Care and Use Committee. The study was conducted in general accordance with (a) applicable regulations of the US FDA Good Laboratory Practice (GLP) Regulations for Nonclinical Laboratory Studies standards, 21 CFR Part 58 (5 October 1987) [34]; (b) study protocol and TRS Labs, Inc. standard operating procedures (SOPs). The randomization and descriptive statistics were conducted by BioSTAT Consultants, Inc. (Mattawan, MI, USA) and clinical pathology assessments were performed at Antech Diagnostics GLP (Morrisville, NC, USA).

### Experimental animals

A total of 24 purebred Beagles (12 male and 12 female) were included in the study. These dogs were at least 8 months of age and had a body weight (BW) range of 7.1–11.4 kgs on study day (SD) –1.

The healthy dogs were surgically-implanted with ten male and ten female adult *D. immitis* worms (Georgia III isolate) in the jugular vein 2–3 months prior to the start of the study, as previously described [35, 36].

### Randomization and treatment

A gender-stratified, randomized, placebo-controlled, blinded, parallel group design was used. Dogs that met the inclusion criterion of good health on SD –6/–7 (as determined by physical examinations and standard clinical pathology parameters) and verified to be heartworm-positive via an antigen test and a MF count of at least 300 MF/mL (measured by modified Knott test), were randomly assigned to three treatment groups containing eight dogs each (four male and four female). Allocation of dogs to individual pens was performed as part of the randomization plan. The randomization plan was created using SAS software (SAS Institute, Cary NC; version 9.4).

**Fig. 1:**
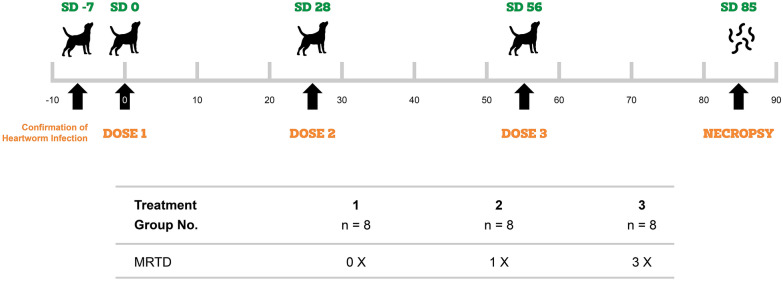
Schematic representation of study schedule. *MRTD* maximum recommended therapeutic dose, *n* number of dogs, *1×* = 40 mg/kg lotilaner + 0.04 mg/kg moxidectin + 10 mg/kg praziquantel + 10 mg/kg pyrantel

According to VICH GL 43 guidelines, the margin of safety is evaluated by considering multiples of the MRTD. The MRTD refers to the dose intended for the lightest weight dog within the broadest dose range [37]. For Credelio Quattro, the MRTD is approximately 40 mg/kg lotilaner + 0.04 mg/kg moxidectin + 10 mg/kg praziquantel + 10 mg/kg pyrantel. In this study, group 1 was the negative control and received 0× (placebo tablet), group 2 received 1×, and group 3 received 3× MRTD of Credelio Quattro. The dogs were dosed orally in the fed state on SD 0, 28, and 56 (schematic depiction of schedule of events in Fig. 1). The test article dose was calculated on the basis of the dog’s most recent body weight, which was obtained the day prior to each treatment administration. As whole tablets were used, point dosing was not possible. A combination of tablets was utilized to get as close as possible to the MRTD. Dogs were not under-dosed by more than 10% of the MRTD. If dosing by less than 10% of the MRTD was not possible, dosing exceeded the maximum required dose. If a dog vomited within 2 h of dosing, it was re-administered a full dose of new tablet(s), with only one re-dose per dose cycle.

### Clinical observations

From SD –7 to the conclusion of the study, individual general health observations (GHOs) were generally conducted twice daily (a.m. and p.m.) at least 6 h apart to evaluate general health, clinical signs of avermectin associated toxicity, and signs associated with death of MF and adult heartworms. Additional observations were conducted on all dogs at approximately 1, 2, 4, 6, 8, 12, and 24 h after each treatment. Physical examinations were performed on SD –6, 27, and 55 (i.e., prior to each treatment) and on SD 84 (prior to necropsy).

### Heartworm diagnostics

The heartworm disease status of each dog was assessed on SD –7 and SD 57 for the presence of *D. immitis* antigen using a commercial heartworm antigen diagnostic test (DiroCHEK^®^ Canine Heartworm Antigen Test Kit (Zoetis, Kalamazoo, MI, USA)). DiroCHEK^®^ is reported to have 100% sensitivity and 100% specificity [38, 39]. MF counts were assessed using the modified Knott test prior to each treatment (SD –7, 27, and 55) and shortly after each treatment (SD 1, 29, and 57).

### Food and water

The dogs had ad libitum access to food during acclimation and on nontreatment days. Fasting occurred prior to clinical pathology evaluation during acclimation and prior to each dosing day. To promote food consumption on treatment days, dogs were fasted overnight (approximately 12 h), followed by presentation of a highly palatable wet-canned food (Nature’s Recipe, LLC; San Fransisco, CA) at a rate equal to approximately 25% of the manufacturer’s recommended daily amount based on body weight. The amount of canned food consumed was evaluated after up to 20 min. If not consumed, the dog was hand fed by placing small amounts of food into the back of the mouth. After oral dosing, the dogs were offered their daily ration of dry food (Teklad Global 21% protein diet (Envigo; Madison, WI)). Dogs had ad libitum access to fresh drinking water on all SDs.

### Necropsy and heartworm counting

Study animals were humanely euthanized with an intravenous injection of euthanasia solution (acepromazine and butorphanol, followed by pentobarbital) combined with 1 mL of heparin solution to facilitate parasite recovery on SD 85, as per the American Veterinary Medical Association Guidelines for Euthanasia (2020) [40]. The abdominal cavity, followed by the thoracic cavities, was opened and examined for any findings and documented. The anterior and caudal vena cava were then clamped with forceps, and the heart and lungs were removed as a unit and placed in a labeled container. The heart and lungs were dissected, and the heartworms retrieved from the vena cava, pre-cava, right atrium, right ventricle, and pulmonary arteries were placed in petri dishes containing saline.

Adult heartworm counts included the sum of the total intact worms (live and dead) per animal. Heartworm fragments were counted as follows: worm fragments containing a head and worm fragments containing a tail were counted separately. The greater of the two counts was included in the worm count. When fragments containing heads or tails were found, any other fragments without heads or tails present did not contribute to the worm count. If only fragments without heads or tails were found, the fragments were collectively considered to represent one worm.

### Statistical analysis

Descriptive statistical analyses of worm counts, MF counts, and health observations were conducted by BioSTAT Consultants. MF counts were transformed using natural logs and analyzed in a general linear model with treatment group, study day, and the interaction between group and day as fixed effects. Sex was included as a random effect, and the correlation between observations on the same dog was accounted for via repeated measures methods. On each study day, MF counts in the control group were compared with those in the other treatment groups. Model means and 95% confidence limits were back-transformed for presentation. Data were analyzed with the statistical software package SAS^®^ (version 9.4, SAS Institute Inc., Cary, NC, USA).

## Results

### Study inclusion

Prior to study initiation, all 24 dogs tested positive for the presence of *D. immitis* antigen, with all dogs far exceeding the 300 MF/mL required for inclusion in the study. The geometric mean MF counts 7 days pre-treatment for group 1 (0×), group 2 (1×), and group 3 (3×) were 8205, 7096, and 8230 MF/mL, respectively.

### Dosing

Actual doses received were very close to the target, thus thoroughly testing the 1× and 3× MRTD. A summary of the mean and standard deviation of doses received across all dose cycles is presented in Table 1.

**Table 1:** Summary of doses received across all dose cycles

Active ingredient	Mean dose received (mg/kg)	
	1×	3×
Lotilaner	39.15 ± 1.355	119.59 ± 1.624
Moxidectin	0.039 ± 0.0014	0.120 ± 0.0016
Praziquantel	9.92 ± 0.343	30.30 ± 0.411
Pyrantel	9.92 ± 0.344	30.30 ± 0.411

Doses are presented as mean ± SD of all dose cycles and only include the initial dose offering (i.e., re-administration owing to emesis not included)

### Clinical observations

There were no meaningful changes in weight in the study animals, with the average weight of the groups remaining similar throughout the study. There were no incidents of emesis in the control (0×) and 1× dogs, whereas 18 incidents of emesis were observed in the 3×, with at least one incident in all 8 dogs. Two dogs in the control group had four incidents of diarrhea, three dogs at 1× had four incidents, and three dogs at 3× had five incidents. Owing to the timing of events relative to dosing, emesis and diarrhea were possibly related to treatment; however, all dogs recovered in a short period of time and without treatment. There were no signs of avermectin toxicity observed in any treated dog. Other clinical observations reported during the study were minor and included lameness, dermatitis, pruritis, and trauma. None of these abnormalities were treatment related.

### Microfilaria count

**Fig. 2:**
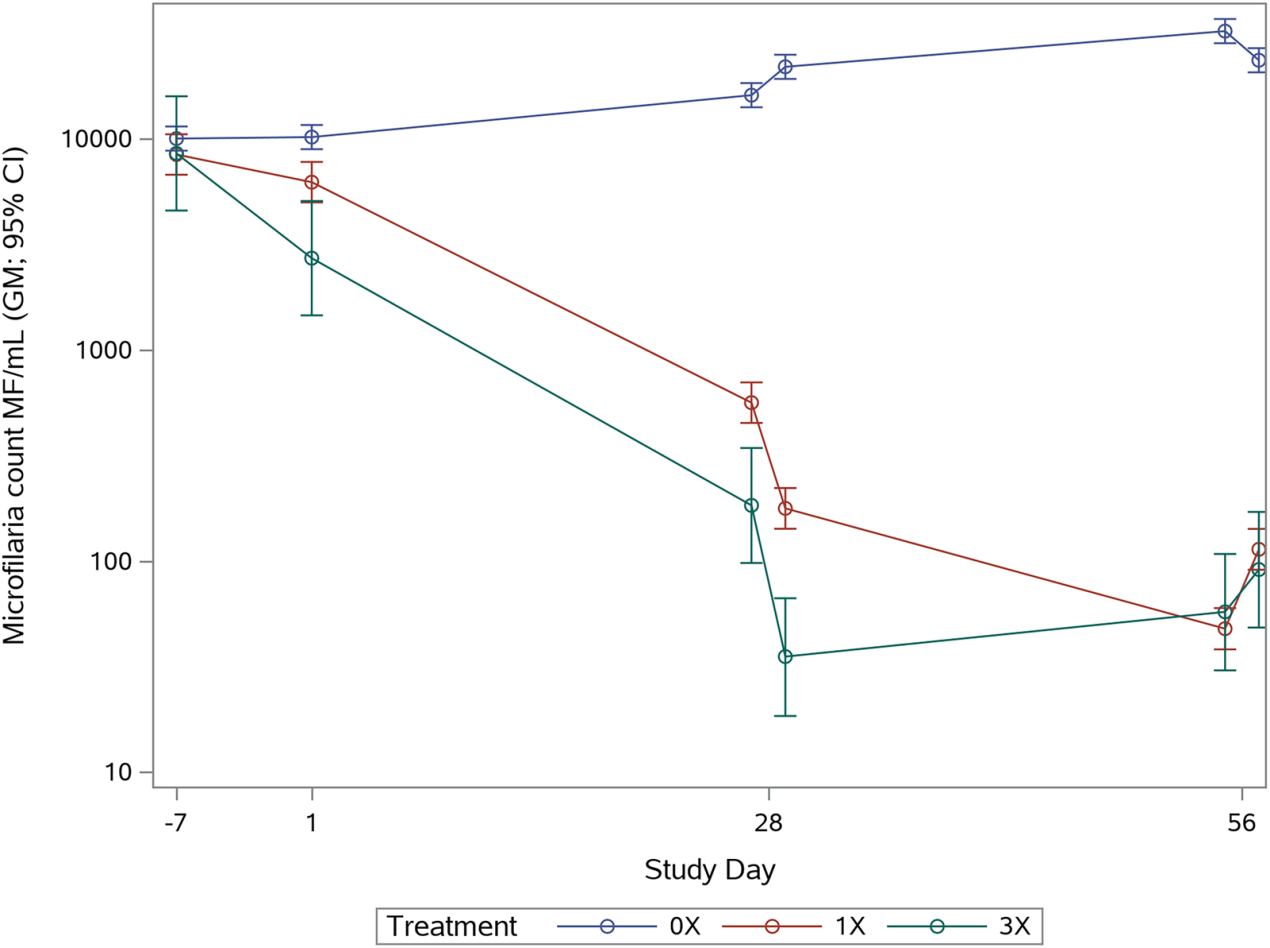
Geometric mean with 95% confidence intervals for MF counts over time. Knott test was performed on Days −7, 1, 27, 29, 55, and 57

The summary of the MF/mL at various time points is depicted in Table 2 and Fig. 2. The geometric means with percent (%) reduction for the treated groups as compared with the control group are provided in Table 3. The geometric mean MF counts started in the control group at 10,045/mL (SD −7), increased to > 16,000/mL in the second dose cycle and to > 20,000/mL for the remainder of the study. On SD 1, the Credelio Quattro treatment was already reducing the amount of circulating MF, as compared with the control group by 38% for the 1× dose and by 73% for the 3× dose. By the end of the first month, both treatment groups had reduced MF counts by > 96%. Following the subsequent doses, the reduction was greater than 99% for both treatment groups.

**Table 2:** Range of blood microfilaria counts (microfilaria/mL) over time

Treatment group	Pre-treatment (study day –7)	Study day 1	Study day 27	Study day 29	Study day 55	Study day 57
Group 1 (0×)	3550–13,400	7000–15,200	12,950–18,400	17,450–25,750	19,100–42,100	15,150–28,100
Group 2 (1×)	3750–10,750	4550–10,200	10–1250	17–400	1–500	5–1150
Group 3 (3×)	5250–14,350	1300–5450	0–1100	0–390	0–215	0–500

**Table 3:** Geometric mean microfilariae count (counts/mL) with % reduction

	Pre-treatment (study day –7)	Study day 1	Study day 27	Study day 29	Study day 55	Study day 57
Control	10,045	10,209	16,129	21,980	32,441	23,609
1×	8442	6243*	563*	178*	48*	113*
% reduction	14.0	38.8	96.5	99.2	99.9	99.5
*P*-values	0.1415 (*t*_125_ = 1.48)	< 0.0001 (*t*_125_ = 4.18)	< 0.0001 (*t*_125_ = 28.53)	< 0.0001 (*t*_125_ = 40.94)	< 0.0001 (*t*_125_ = 55.29)	< 0.0001 (*t*_125_ = 45.32)
3×	8544	2724*	184*	35*	57*	91*
% reduction	14.9	73.3	98.9	99.8	99.8	99.6
*P*-values	0.6129 (*t*_125_ = 0.51)	0.0321 (*t*_125_ = 4.14)	< 0.0001 (*t*_125_ = 14.01)	< 0.0001 (*t*_125_ = 20.07)	< 0.0001 (*t*_125_ = 19.8)	< 0.0001 (*t*_125_ = 17.38)

^*^statistically different (*P* ≤ 0.05)

### Adult heartworm counts

Heartworms recovered and counted at necropsy at the end of the study (SD 85), are depicted in Table 4. No dead adult worms were found in any group. The mean number of implanted adult worms recovered was < 10 per sex in all groups with 87.5%, 94.5%, and 79.5% of live adult heartworms recovered in groups 0×, 1×, and 3×, respectively. The mean adult heartworm count for group 3 (3×) was numerically lower than the control group owing to natural variation, and there was no evidence of effect of the test article on adult worms.

**Table 4:** Mean ± SD of total number of adult heartworms recovered at necropsy on study day 85

	Male		Female		Fragments containing		
	Alive	Dead	Alive	Dead	Head	Tail	Total
Group 1 (0×)	8.9 ± 1.13	0.0	7.8 ± 2.05	0.0	0.4 ± 0.74	0.6 ± 0.92	17.5 ± 2.07
Group 2 (1×)	8.6 ± 1.19	0.0	8.9 ± 1.25	0.0	0.8 ± 1.04	0.9 ± 1.46	18.9 ± 1.25
Group 3 (3×)	7.9 ± 1.36	0.0	7.4 ± 0.92	0.0	0.1 ± 0.35	0.4 ± 0.52	15.9 ± 1.89

## Discussion

The safety of Credelio Quattro in dogs with adult *D. immitis* and with circulating MF was evaluated in this study over the course of three consecutive administrations 28 days apart. A single dose of Credelio Quattro in the commercially available formulation, when used according to the label, delivers a dose of moxidectin between 20 and 40 μg/kg. When the maximum dose was tested at 1× and 3× multiples in heartworm-positive dogs, the dose was well tolerated, with treatment related observations limited to emesis (3× group only) and occasional abnormal stools (e.g., diarrhea), which were observed shortly after dose administration and resolved without treatment. Emesis at 3× has been reported in normal healthy Beagle dogs, with vomiting increasing with dose [41]. As dogs that experienced emesis within the first 2 h of dose administration were re-administered another full dose, there was ample opportunity for the drug to be absorbed and the potential for dogs receiving the 3× dose to have achieved closer to a 6× dose. Therefore, emesis at 3× did not impact the assessment of safety in this study.

### Microfilaricidal activity

On SD 1, Credelio Quattro reduced the amount of circulating MF (as compared with the control group) by 38.8% for the 40 µg/kg (1×) dose and significantly reduced the amount by 73.3% for the 120 µg/kg (3×) dose (Table 3). By the next evaluation time point (end of the first month, SD 27), both treatment groups had significantly reduced the MF count by > 96% as compared with the concurrent control. Following subsequent doses, the reduction remained statistically different from the control group and was greater than 99% for both treatment groups. Despite the MF count sharply decreasing in both 1× and 3× groups compared with control post-treatment, there were no associated abnormalities noted due to death of microfilaria or worms and no hypersensitivity reactions (anaphylaxis, shock, collapse, respiratory distress, or depression).

Similarly designed and conducted studies were completed with NexGard Plus^®^ (Boehringer Ingelheim, Duluth, GA, USA) and Simparica Trio^®^ (Zoetis, Kalamazoo, MI, USA). In the NexGard Plus study, the doses of moxidectin tested were 24 µg/kg MRTD (1×) and 72 µg/kg MRTD (3×) [42]. Similar to Credelio Quattro, the only clinical observations noted for NexGard Plus were diarrhea and emesis. Simparica Trio was reported to cause fever in two heartworm-positive dogs approximately 24 h after dosing (one dog in the 1× MRTD group (48 µg/kg moxidectin) and one dog in the 3× MRTD group (144 µg/kg moxidectin)) [43]. It was hypothesized that the fever was caused by the rapid reduction of MF in these two dogs. Importantly, fever was not observed in any dogs following the administration of Credelio Quattro (moxidectin doses of 40 µg/kg in 1× and 120 µg/kg in 3× MRTD).

All three products demonstrate a rapid rate of MF clearance, with NexGard Plus reducing circulating MF by 94%, Simparica Trio by > 99%, and Credelio Quattro by 97% by the end of the first month of treatment at their respective 1× MRTDs as compared with the control group [42, 43]. NexGard Plus reduced MF by 97.4% following subsequent doses, whereas Simparica Trio and Credelio Quattro were > 99%.

### Adulticidal activity

In this study, no dead worms were observed in any treatment group. Furthermore, there was no evidence of adulticidal activity with Credelio Quattro-treated groups having similar total worm counts as the control group. Importantly, there were no clinical signs attributable to the death of adult heartworms in this study (e.g., cough, dyspnea, exercise intolerance, collapse, hemoptysis, and/or death).

## Conclusions

Credelio Quattro, when administered at 1× and 3× the maximum recommended label dose, was well tolerated following three consecutive monthly administrations (28-day intervals) to dogs that had been previously infected with adult *D. immitis*. Although Credelio Quattro caused a rapid reduction in MF counts in both treatment groups, no adverse effects related to MF reduction were observed, and there was no effect on adult worms in this study.

## Data Availability

Datasets generated and/or analyzed during the current study are not publicly available owing to commercial confidentiality of the research. Data not included in the manuscript can only be made available to bona fide researchers subject to a fully executed nondisclosure agreement.

## Abbreviations

ARRIVE: Animal research reporting in vitro experiments
CFR: Code of federal regulations
FDA: (US) Food and Drug Administration
GLP: Good laboratory practice
Inc.: Incorporated
MF: Microfilaria
ML: Macrocyclic lactone
MRTD: Maximum recommended therapeutic dose
OECD: Organization for economic cooperation and development
SAS: Statistical analysis system
SOPs: Standard operating procedures
TRS: TRS Labs, Inc.
USA: United States of America
VICH: Veterinary International Conference on Harmonization
